# Autoantibodies Against Albumin in Patients With Systemic Lupus Erythematosus

**DOI:** 10.3389/fimmu.2018.02090

**Published:** 2018-10-02

**Authors:** Josephine Nehring, Lucia A. Schirmbeck, Justa Friebus-Kardash, Denise Dubler, Uyen Huynh-Do, Carlo Chizzolini, Camillo Ribi, Marten Trendelenburg

**Affiliations:** ^1^Division of Internal Medicine and Clinical Immunology laboratory, Department of Biomedicine, University Hospital Basel, University of Basel, Basel, Switzerland; ^2^Department of Nephrology, University Hospital, Essen, Germany; ^3^Department of Biomedicine, University of Basel, Basel, Switzerland; ^4^Nephrology and Hypertension, University Hospital Bern, Bern, Switzerland; ^5^Internal Medicine Specialties, Clinical Immunology and Allergy, Geneva University Hospitals, Geneva, Switzerland; ^6^Department of Immunology and Allergy, Lausanne University Hospital, Lausanne, Switzerland

**Keywords:** antibodies, human serum albumin, patients, systemic lupus erythematosus, multicenter study

## Abstract

**Objectives:** Autoantibodies and aberrant immune complexes are pathological hallmarks of systemic lupus erythematosus (SLE). This study aimed to determine the occurrence of IgG autoantibodies against human serum albumin (anti-HSA IgG) and their potential association with antibodies against bovine serum albumin (anti-BSA IgG) in patients with SLE.

**Methods:** Sera of 180 SLE patients included to the Swiss SLE Cohort Study and 188 age- and sex-matched healthy controls were evaluated. Levels of anti-HSA IgG and anti-BSA IgG were quantified by ELISA. Selected samples were further characterized using serum fractions obtained by fast liquid chromatography (FPLC).

**Results:** SLE patients had increased levels of anti-HSA IgG (*p* = 0.002) but similar levels of anti-BSA IgG compared to matched healthy controls. Anti-HSA IgG levels correlated with the SLE Disease Activity Index (SLEDAI), which was more pronounced in patients with an physician's global assessment (PGA) of ≥ 1 (*r* = 0.309, *p* = 0.0066). Anti-HSA IgG was partially complexed with serum albumin but also occurred as monomeric autoantibodies in highly positive SLE patients. A positive correlation between anti-HSA IgG and anti-BSA IgG was found that was stronger in SLE patients than in healthy controls (*r* = 0.3172, *p* < 0.001 vs. *r* = 0.2122, *p* < 0.0035). Binding of anti-BSA IgG was inhibited partially in the presence of HSA in samples with double positivity for anti-HSA and anti-BSA (median inhibition 47.9%, range 0.9–100%) and vice versa.

**Conclusion:** In SLE patients there is an increased prevalence of anti-HSA IgG antibodies that are associated with SLE disease activity.

## Introduction

Systemic lupus erythematosus (SLE), particularly frequent in women of childbearing age, has a highly variable clinical presentation with flares and remissions. Pathogenic mechanisms leading to SLE are still not fully understood. A hallmark of SLE is the occurrence of a broad spectrum of antibodies targeting self-antigens. Although it still remains unclear how ubiquitous molecules become autoantigens, there is good evidence that autoantibody production in SLE is antigen driven (1). One major but not exclusive hypothesis is that apoptotic cells and defects in their clearance are the origin for autoantigens and thus a trigger for the autoimmune response. Apoptotic cells are identified, taken up and degraded by professional and/or neighboring phagocytes (2). The mechanisms underlying the recognition and engulfment of these cells involves many receptors, bridging molecules, and signal cascades among which complement seems to play a prominent role (3). A defective clearance of apoptotic material might induce an aberrant immune response leading to the generation of a large number of autoantibodies (4).

So far, SLE is the autoimmune disease with the largest diversity of detectable autoantibodies with more than 180 different specificities having been described, but only a few have been shown to be directly involved in tissue injury (5–7). However, published investigations on human's most abundant protein in plasma, i.e., albumin, as an autoantigen are scarce. As a target of autoantibodies, albumin would not obviously fit with the hypothesis of an impaired clearance of apoptotic cells as a trigger for the development of SLE. While albumin was found to protect human endothelial cells from apoptosis, an albumin overload has been shown to induce apoptosis in renal tubular cells (8–12).

Functionally, in spite of only two major binding sites albumin shows diverse affinities, e.g., for hydrophobic ligands like fatty acids, bilirubin, trace elements, hormones or pharmaceutical substances (13). Though albumin plays major physiological roles (carrier protein, regulation of blood pressure, stabilization of blood pH etc.), rare cases of individuals were described that essentially lacked albumin and exhibited only minor symptoms, thus questioning the importance of the molecule (13). With regard to a potential origin of autoantibodies against HSA, it is of interest to note that HSA shares almost 80% sequence identity with bovine serum albumin (BSA), which is present in western diet (14). Previous studies (15–17) focused on the possibility of BSA to be a causative agent for the onset of autoimmune diseases, but the definite role of BSA in autoimmunity remains to be established. More specifically, cross-reactivity between BSA and human autoantigens, e.g., collagen type I, vitamin D binding protein, myelin basic protein and islet beta cells, has been reported in patients with rheumatoid arthritis, multiple sclerosis, and diabetes mellitus. Furthermore, a more recent study described a pathogenic role for cationic BSA in patients with early-childhood membranous nephropathy (18). As for anti-HSA, data on anti-BSA in patients with SLE are scarce.

Therefore, our study aimed to assess the occurrence of autoantibodies against human serum albumin (anti-HSA) and their potential association with antibodies against bovine serum albumin (anti-BSA) in a large and well-defined cohort of SLE patients compared to age- and sex-matched healthy controls.

## Materials and methods

### Patients and clinical data

Blood samples of 180 patients suffering from SLE and fulfilling at least 4 of the 11 classification criteria of the American College of Rheumatology (ACR) were provided by the Swiss Systemic lupus erythematosus Cohort Study (SSCS) (19). The samples were consecutively collected between 2008 and 2013 at the University Hospitals in Basel, Bern, Geneva, and Lausanne (all in Switzerland). The collection of clinical data followed a standardized form and was recorded anonymously in an online database after having obtained written informed consent from all participating patients and clearance from the Ethical Committees of the involved institutions. In our cross-sectional study, only samples collected at the first study visit were analyzed.

The SLE patients had a median age of 42 years (range 16–84, 85% female), a median disease duration of 6 years since diagnosis and a median SLE Disease Activity Index (SLEDAI) score of 4 at the time of blood sampling. The patient characteristics are summarized in Table 1.

**Table 1:** Characteristics of 180 patients with systemic lupus erythematosus.

**Number of patients, n**	**180**
Female, *n* (%)	153 (85)
Male, *n* (%)	27 (15)
Age, median (range)	42 (16–84)
Disease duration at inclusion time, median (range)	6 (0-52.17)
Anti-dsDNA antibodies positive, *n* (%)	161 (92.8)
Complement C3 (g/L), median (range) (norm value 0.8–1.8 g/L)	0.73 (0.27–1.95)
Complement C4 (g/L), median (range) (norm value 0.1–0.4 g/L)	0.11 (0.02–0.47)
SLEDAI, median (range)	4 (0–38)
SLICC-SDI, median (range)	0 (0–9)
History of nephritis, *n* (%)	56 (31)
History of arthritis, *n* (%)	41 (23)
Systemic corticosteroids, *n* (%)	109 (61)
Antimalarial agents, *n* (%)	113 (63)
Immunosuppressant agents, *n* (%)	80 (44)

*Anti-dsDNA, anti-double stranded deoxyrubinucleic acid antibodies; SLEDAI, SLE Disease Activity Index; SLICC-SDI, Systemic Lupus International Collaborating Clinics-SLE Damage Index*.

Serum specimens were also obtained from 188 age- and gender-matched healthy blood donors having a median age of 49 years (range 19–81, 85.6% female). Blood samples from patients and healthy controls were aliquoted and frozen at −80°C until further use.

Inclusion criteria of SLE patients for this study were: Age ≥ 18 years with at least 4 classification criteria according to the American College of Rheumatology (ACR), complete clinical data for judgement of disease activity, availability of blood samples and signed informed consent. There were no specific exclusion criteria. The clinical manifestations were assessed in accordance with the SLEDAI.

SSCS was approved by the responsible ethical committees of all contributing centers.

### Antigens and detection of antibodies

Antibodies were detected by ELISA using ninety-six well plates (Maxi sorp, Thermo Denmark) that were coated with HSA and BSA as antigen respectively [both from Sigma-Aldrich USA; HSA: A8763, i.e., ≥99% pure and essentially free of globulins; BSA: A7906 and A7030 (protease free, fatty acid free, essentially globulin free)] at a concentration of 2 μg/ml diluted in coating buffer (0.2 M Carbonate buffer pH 9.6) overnight at 4°C.

After washing with Phosphate Buffered Saline (PBS, homemade) containing 0.05% Tween 20 (Sigma-Aldrich, USA) (PBST), we applied patient and healthy control sera (centrifuged for 30 min at 4°C at 2,1000 g) diluted 1/50 either in PBS Tween 20 (containing 0.14 M NaCl, Gibco) or in PBS Tween 20 containing 1 M NaCl (in order to reduce low affinity binding) in duplicates followed by an incubation at room temperature (RT) for 1 h on a plate shaker (Sarstedt TPM-2, 400 rpm).

Bound antibodies were detected with a secondary goat anti-human IgG (Fc gamma) specific antibody conjugated with alkaline phosphatase (Jackson ImmunoResearch) followed by alkaline phosphatase substrate (Sigma-Aldrich). The enzymatic reaction was quantified after 20 min at 405 nm by a microplate biokinetics reader (BioTEK Instruments, USA).

Anti-BSA antibody levels were quantified in relative Units (rU) based on serial dilutions of one serum of the healthy donor “MO” having been found to have high levels of monomeric IgG (see below) with high avidity for BSA (K_D_ value = 6.45 × 10^−10^ as estimated by Scatchard plot analysis adapted to the method described by Hulme et al. [(20), data not shown]. A dilution of 1/100 was considered to be equivalent to 1000 relative Units (rU). Because of a lack of standard for the determination of anti-HSA levels, only few positive signals for anti-HSA IgG in sera of both groups, a similar antigen (HSA vs. BSA), and the use of the same secondary antibody for the determination of anti-HSA IgG and anti-BSA IgG respectively, we decided to also use the anti-BSA IgG standard for the calculation of anti-HSA IgG levels.

Anti-BSA IgG and anti-HSA IgG levels <1 rU corresponded to the background of the assay and thus were considered to be negative. This approach was chosen because of a lack of normal distribution (not allowing a proper use of standard deviations) and because of the possibility that normal individuals could be positive for anti-albumin antibodies as well.

As a control, in order to exclude the presence of DNA/nucleosomes as a potential source of falsely positive signals (in spite of the high purity of the HSA preparation), samples being positive for the presence of anti-HSA IgG were also tested on HSA that was pretreated for 1 h at RT with increasing amounts of DNAse up to a maximum of 100 μg/ml (DNAse I from bovine pancreas, Sigma-Aldrich USA, D5025).

Moreover, samples from two SLE patients and one healthy control being positive for anti-HSA on ELISA were also investigated by Western blot using purified reduced and unreduced HSA as antigen (Sigma-Aldrich USA, A8763). In short, reduced/unreduced albumin was loaded on a 12% SDS Tris-Glycine gel (Biorad USA, 4561044), then transferred on a nitrocellulose membrane (Biorad USA, 1620115) and blocked using protein free blocking buffer TBS+0.1% Tween 20. Then, sera diluted 1:200 in TBS+0.1% Tween 20 were incubated for 1 h at RT and, after washing, bound IgG was detected using a goat anti-human IgG (Fc gamma) specific antibody conjugated with horseradish peroxidase (Jackson ImmunoResearch USA, 109-035-008).

### Characterization of anti-HSA in a subgroup of SLE patients and healthy donors

To determine whether the detected anti-HSA IgG were monomeric or complexed IgG, we fractionated serum of the two SLE patients and serum of the healthy control with the highest antibody levels by fast protein liquid chromatography (FPLC, GE ÄKTApure 25 L1, Sweden) based on protein size using a Superdex 200 Increase 10/300 GL column (GE, Sweden). The fraction size was 0.4 ml. All fractions then were analyzed for the presence of anti-HSA IgG by ELISA as described before.

As our anti-HSA IgG ELISA might only detect free anti-HSA IgG antibodies but miss those antibodies that have already bound to serum albumin, the serum fractions of patients as well as of matched controls were also tested for the presence of albumin (HSA)-IgG complexes. For this, we established a “sandwich” ELISA using a goat polyclonal anti-HSA antibody (Abcam, ab19180 anti-human serum albumin antibody) as catching antibody and a AP-labeled goat anti-human-Fc gamma chain (Jackson, 109-055-008) as detecting antibody. Areas under the curve (AUC) of albumin (HSA)-IgG were calculated in fractions positive for these complexes. Selected samples of SLE patients (*n* = 8) and healthy controls (*n* = 8) matched for total anti-HSA IgG and anti-BSA IgG levels were used to determine potential differences between patients and controls.

Last, having detected albumin (HSA)-IgG complexes by ELISA in serum fractions that normally should not contain albumin (i.e., having a molecular weight of > 100 kD), the presence of albumin in these fractions was verified by Western blot. For this, FPLC fractions of anti-HSA IgG positive individuals (one patient and one donor) containing molecules with an estimated molecular weight of >100 kD were loaded on a 12% SDS Tris-Glycine gel (Biorad USA, 4561044) (reduced and unreduced), and then transferred on a nitrocellulose membrane (Biorad, 1620115). Albumin was detected by a combination of a goat anti-human serum albumin antibody (Abcam UK, ab 19180) followed by a HRP-labeled monoclonal anti-goat/sheep IgG antibody (Sigma, A9452).

### Inhibition of antibody binding by the presence of fluid phase HSA and BSA

To analyze a potential cross-reactivity between anti-HSA IgG and anti-BSA IgG, samples with double positivity (*n* = 27 for SLE patients; *n* = 13 for healthy controls) were analyzed in the presence or absence of an excess of fluid phase BSA or HSA. Differences in signal intensity recorded in the presence of fluid phase antigen, were considered to reflect cross-reactivity and expressed as percentage of response according to the formula: 100/rU of plate-bound antibody × rU of bound antibody in presence of fluid phase antigen.

### Statistical analysis

All statistical analyses except the calculation of areas under the curve (AUC) were performed using GraphPad Prism Version 7. The calculation of AUC was performed by program R (package “pracma”).

Because of an asymmetric distribution, data are expressed as median with range. Differences between two groups were analyzed by two-tailed Mann-Whitney test or Fisher exact test for unrelated data and Wilcoxon matched-pairs signed rank test for matched samples. Correlations were analyzed by Spearman's rank correlation coefficient. *P*-values below 0.05 were considered to be statistically significant.

## Results

### Antibodies against albumin (anti-HSA IgG) in SLE patients and healthy controls

**Figure 1:**
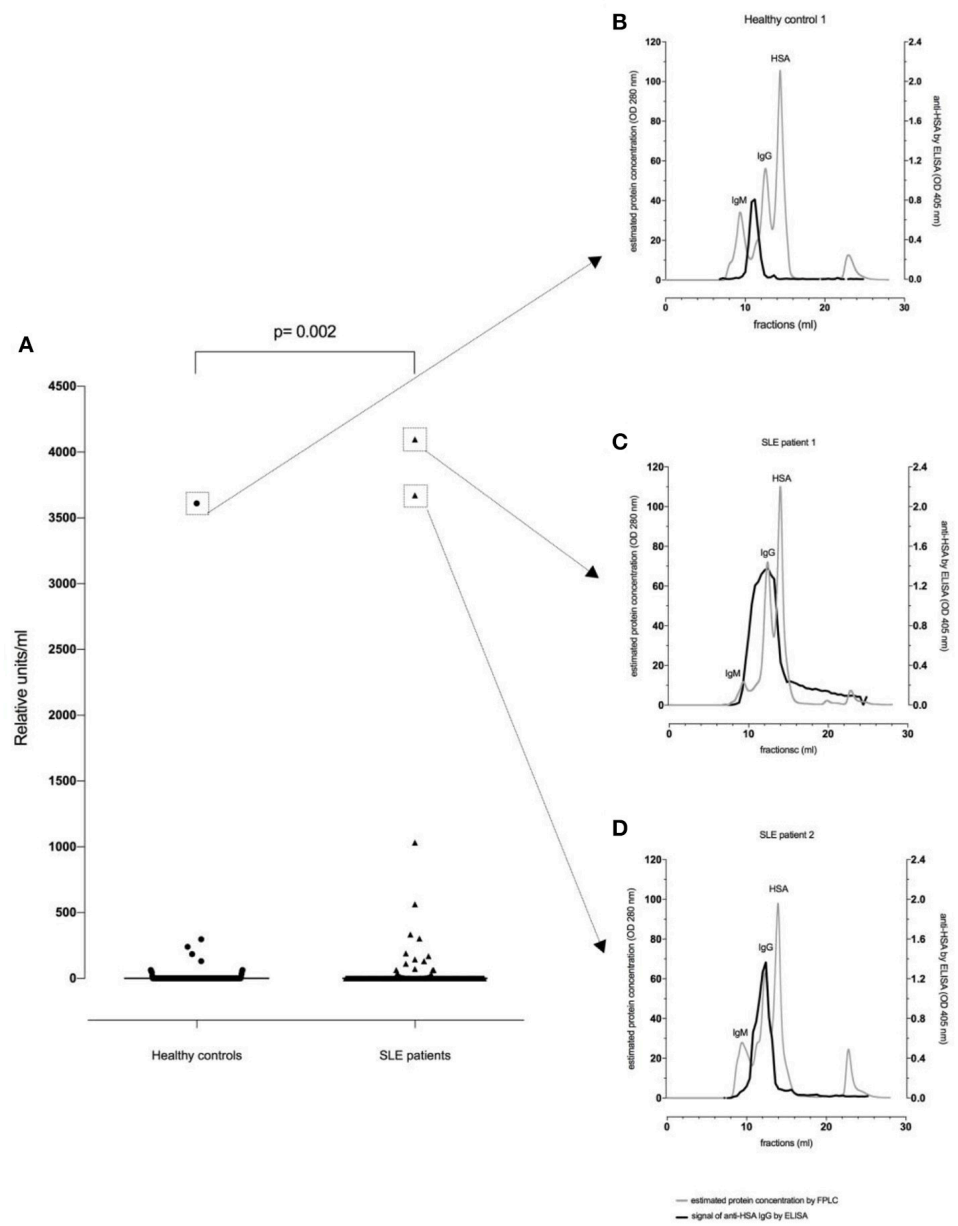
Anti-HSA IgG levels in healthy controls (*n* = 188) and SLE patients (*n* = 180). There was a significant difference between healthy controls and SLE patients regarding **(A)** anti-HSA IgG (*p* = 0.002). FPLC serum profiles and fractions using FPLC were tested by anti-HSA IgG antibody ELISA for the healthy control with the highest anti-HSA IgG level **(B)**, the SLE patient with the highest anti-HSA IgG level **(C)** and for the SLE patient with the second highest anti-HSA IgG level **(D)**.

Thirty-two of the 180 SLE patients (17.8%) and 13/188 healthy controls (6.9%) were found to be positive for anti-HSA IgG. The difference in the frequency of positive samples between SLE patients and healthy controls was significant (*p* = 0.002, Fisher exact test), and antibody levels in SLE patients were significantly higher than in age- and sex-matched healthy controls (*p* = 0.002, Mann–Whitney, Figure 1A).

Pretreatment of HSA with increasing concentrations of DNAse up to 100 μg/ml led to a maximum drop of signal intensity of 21% in one of the SLE sera compared to 7% in sera of healthy controls suggesting that DNA-containing material was not a major confounder in our assay. Furthermore, anti-HSA IgG levels did not correlate with total-IgG levels, neither in SLE patients (*r* = −0.004034; *p* = 0.9571) nor in healthy controls (*r* = −0.04186; *p* = 0.5684) suggesting that the occurrence of anti-HSA IgG did not reflect polyclonal B-cell activation.

### Characterization of anti-HSA

We next sought to determine whether anti-HSA IgG in serum were monomeric or complexed. For this, separate FPLC fractions of the individuals with highest anti-HSA IgG levels were analyzed for the occurrence of anti-HSA IgG allowing an estimation of the size of the molecules leading to a positive test result.

Figure 1B illustrates a representative FPLC protein profile of a healthy control serum (gray line). After incubation of the collected fractions on an anti-HSA IgG ELISA, a signal peak among molecules with an apparent size of about 900 kDa (e.g., IgM, complement C1q) could be observed (black line) indicating that IgG antibodies reactive with HSA were either polymers and/or complexed with antigen (possibly albumin-containing immune complexes).

In contrast, as shown in Figures 1C,D, SLE patients showed an additional signal overlapping with the monomeric IgG peak. Thus, in SLE patients antibodies recognizing HSA can be found in larger complexes but also in smaller complexes and as monomeric IgG.

**Figure 2:**
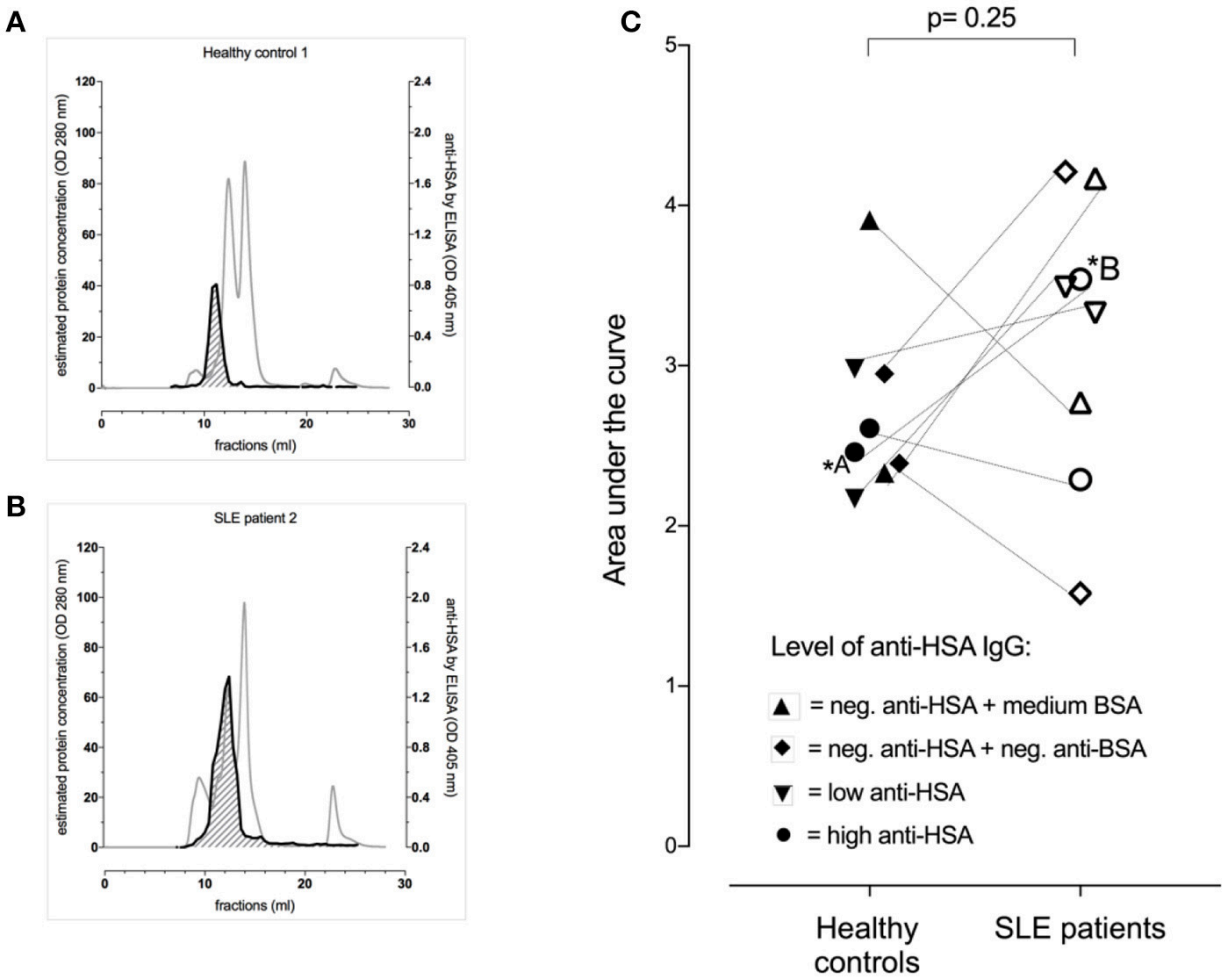
Occurrence of albumin-IgG complexes in representative serum profiles (gray lines) after separation by FPLC of one of the healthy controls **(A)** and the corresponding (matched for anti-BSA IgG and anti-HSA IgG levels) SLE patient **(B)**. Both had high levels of anti-BSA IgG and anti-HSA IgG. The dark line indicated the signal intensity for albumin (HSA)-IgG complexes with the area under the curve (AUC) being shaded (AUC of the healthy control = 2.60 vs. AUC of the matched SLE patient = 4.21). **(C)** Demonstrates pooled data of 8 SLE patients and 8 healthy controls being matched for anti-HSA and anti-BSA levels as highlighted by the use of different symbols. Overall, there was no significant difference between the SLE patients and their corresponding healthy controls, but the SLE patients tended to have more albumin (HSA)-IgG immune complexes.

As this data suggested the presence of albumin-IgG complexes in anti-HSA IgG positive sera, we next aimed to get an estimation of the levels of albumin (HSA)-IgG complexes in selected sera of patients vs. controls matched for anti-BSA IgG and anti-HSA IgG levels. Figures 2A,B show representative profiles of albumin (HSA)-IgG complexes in one SLE patient (Figure 2B, dark line) and it's control (Figure 2A, dark line). Interestingly, SLE patients (*n* = 8) tended to have more albumin (HSA)-IgG immune complexes than healthy controls (*n* = 8), but had not significantly increased overall levels of albumin (HSA)-IgG complexes compared to matched controls (Figure 2C, *p* = 0.25), not even after normalization for either the peak IgG value, or the peak HSA value or both (data not shown). Albumin (HSA)-IgG complexes were also found in sera being negative for (free) anti-albumin antibodies.

In order to verify the presence of albumin in FPLC serum fractions containing molecules with an estimated molecular weight of >100 kD, those fractions of two representative sera (one SLE patient and one control) were analyzed for the presence of albumin by Western blot. In line with the ELISA data shown in Figure 2 and as shown in Supplement Figures 1A–C, albumin was detected in a high molecular weight range (in analogy to the FPLC fractions) when samples were unreduced while reduction of the samples led to a monomeric albumin band in the range of 70 kD.

Last, samples from two SLE patients and one healthy control being positive for anti-HSA IgG by ELISA were also investigated by Western Blot using purified reduced and unreduced HSA as antigen. All three samples tested positive for IgG anti-HSA on reduced HSA but not on unreduced albumin suggesting that conformational epitopes on albumin are of critical importance (Supplement Figure 2).

### Association of anti-HSA IgG with clinical and biological manifestations of SLE patients

**Figure 3:**
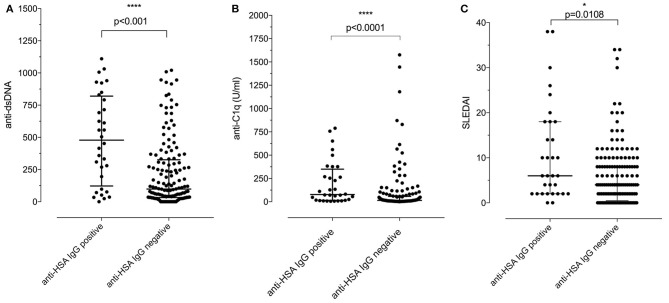
**(A)** Association of positivity/negativity for anti-HSA IgG with levels of anti-dsDNA antibodies in SLE patients. Anti-HSA IgG positive patients had significantly higher levels of anti-dsDNA than anti-HSA IgG negative SLE patients [median of anti-HSA positive: 478 vs. median of anti-HSA IgG negative: 98.5; *p* < 0.001 (Mann–Whitney)]. **(B)** Association of positivity/negativity for anti-HSA IgG with levels of anti-C1q antibodies in SLE patients. Anti-HSA IgG positive patients had significantly higher levels of anti-C1q than anti-HSA IgG negative SLE patients [median of anti-HSA positive: 77.55 vs. median of anti-HSA negative: 14.9; *p* < 0.001 (Mann–Whitney)]. **(C)** Association of positivity/negativity for anti-HSA IgG with the SLEDAI in SLE patients. Anti-HSA IgG positive patients had significantly higher SLEDAI than anti-HSA IgG negative SLE patients [median of anti-HSA positive: 6 vs. median of anti-HSA negative: 4; *p* = 0.0108 (Mann–Whitney)].

We then addressed the question whether the presence of anti-HSA IgG was associated with clinical and laboratory features of SLE. As summarized in Table 2, no associations of anti-HSA IgG were found with age, gender, history of lupus nephritis and central nervous system involvement, respectively. However, anti-HSA IgG were weakly associated with the occurrence of arthritis (*p* = 0.011) and weakly correlated with overall disease activity (*r* = 0.1858; *p* = 0.0128). This correlation was more pronounced in patients with active disease as defined by a Physician's Global Assessment (PGA) ≥ 1 (*r* = 0.309; *p* = 0.0066; *n* = 76). Furthermore, anti-HSA antibodies were found to correlate with anti-dsDNA antibodies (*r* = 0.3119; *p* < 0.0001) and anti-C1q antibodies (*r* = 0.2826; *p* = 0.0001) while no association was observed with levels of complement C3 or C4 respectively. Comparing SLE patients being positive for anti-HSA IgG with those being negative, we found that anti-HSA IgG positive patients had significantly higher levels of anti-dsDNA antibodies (Figure 3A), anti-C1q antibodies (Figure 3B) and SLEDAI scores (Figure 3C).

**Table 2:** Association and correlation of anti-HSA IgG.

**Association of anti-HSA IgG with:**	**Median AU of anti-HSA IgG (range)**	***p*-value**
**Anti-dsDNA antibodies**		ns
yes	0 (0–4097)	
no	0 (0–3)	
**History of nephritis**		ns
yes	0 (0–1033)	
no	0 (0–4097)	
**CNS manifestations**		ns
yes	0 (0–563.5)	
no	0 (0–4097)	
**Correlation of anti-HSA IgG with:**	**Spearman r**	***p*****-value**
Complement C3	*r* = −0.052	ns
Complement C4	*r* = −0.004	ns
Anti-C1q	*r* = 0.283	*p* = 0.0001
Anti-dsDNA	*r* = 0.312	*p* < 0.0001
SLEDAI (all patients, *n* = 180)	*r* = 0.186	*p* = 0.0128
SLEDAI with PGA≥1 (*n* = 76)	*r* = 0.309	*p* = 0.0066
Total IgG	*r* = −0.004	ns

*AU, arbitrary units; CNS, central nervous system; PGA, Physician's Global Assessment; IgG, Immunoglobulin G. Anti-HSA levels <1 rU were considered to correspond to the background of the assay and were set to 0*.

### Association of anti-HSA IgG with antibodies against bovine serum albumin (anti-BSA IgG)

**Figure 4:**
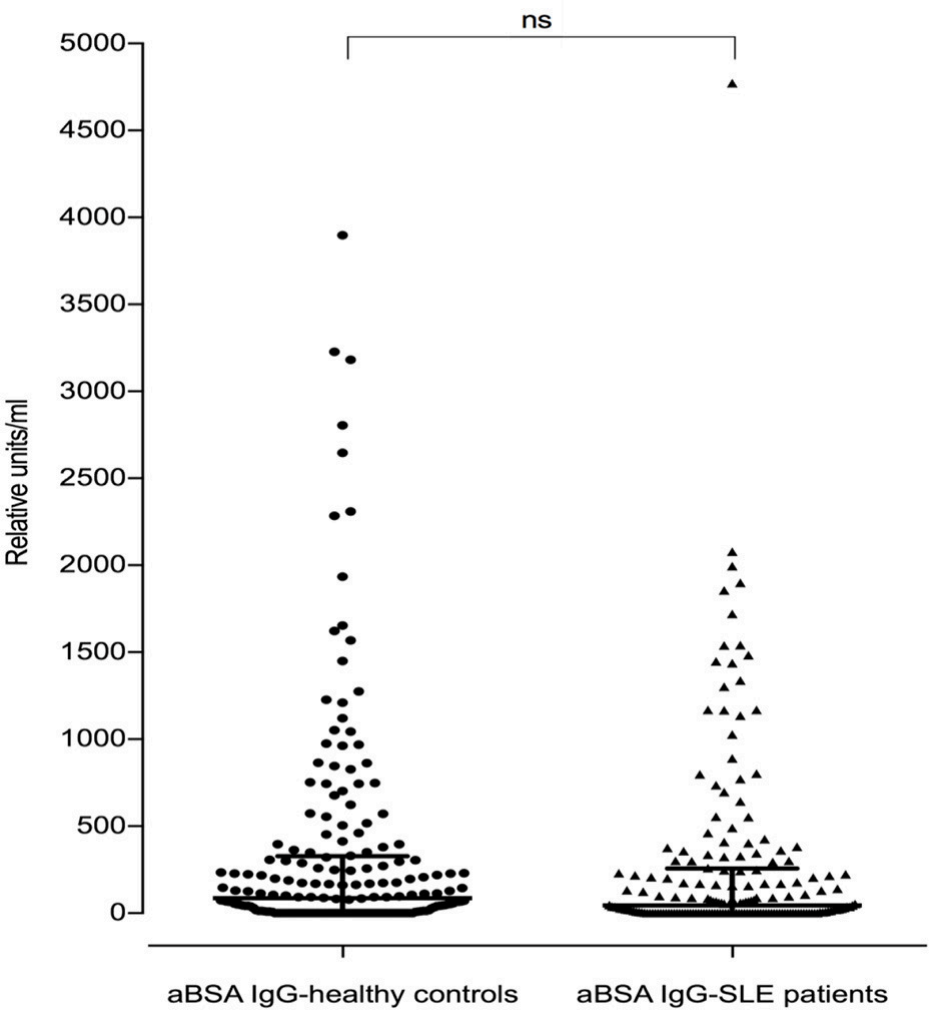
Antibodies against bovine serum albumin in SLE patients (*n* = 180) and healthy controls (*n* = 188). There was no difference seen between SLE patients and healthy controls regarding anti-BSA IgG concentration (*p* = 0.07, two-tailed Mann–Whitney). Bars represent median values.

Of the 180 SLE patients tested, 117 (65%) were positive for anti-BSA IgG vs. 138 of the 188 healthy controls (73.4%) (median in SLE: 45.4 rU vs. median in healthy controls: 86.8 rU). These differences were not significant (*p* = 0.0738, Figure 4).

As for anti-HSA IgG, no correlation of anti-BSA IgG with total IgG was found (SLE patients: *r* = 0.08795; *p* = 0.2404, healthy controls: *r* = 0.0974; *p* = 0.1733). Moreover, no correlation was found between anti-BSA IgG and anti-dsDNA antibody levels in SLE patients (*r* = 0.1438, *p* = 0.0541), and an only weak correlation could be observed between anti-BSA IgG and anti-C1q antibody levels (*r* = 0.1598, *p* = 0.0322).

**Figure 5:**
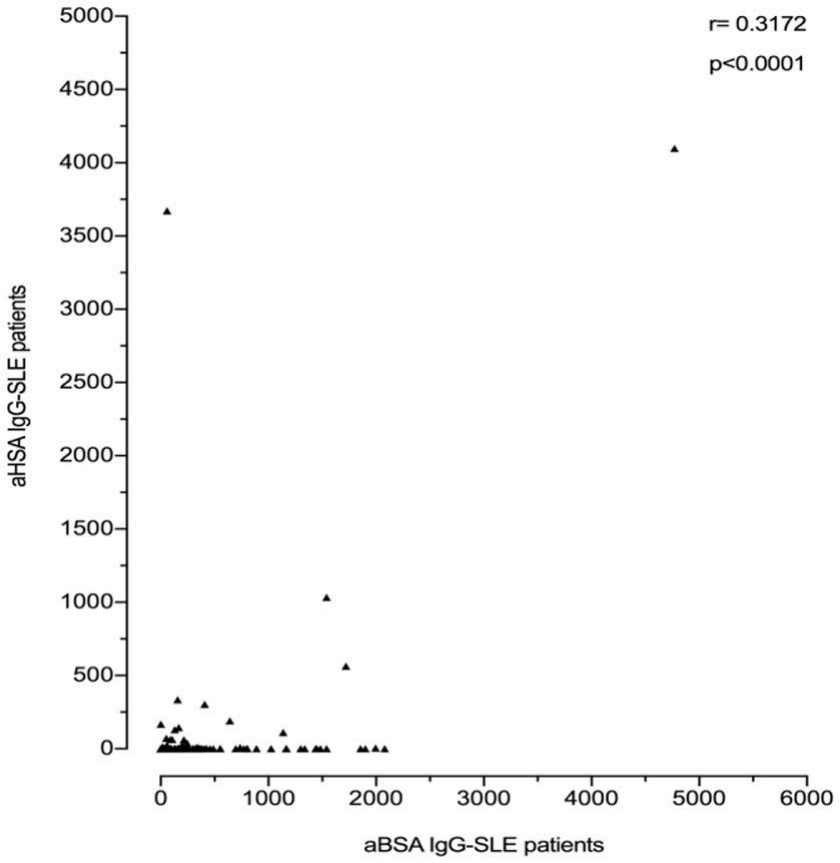
Correlation of anti-BSA IgG level and anti-HSA IgG level in all SLE patients (*n* = 180). A significant correlation was seen between SLE patients and healthy controls with a correlation coefficient *r* = 0.3172 by Spearman's rank correlation (*p* < 0.0001).

However, anti-BSA IgG correlated with anti-HSA IgG levels. This was more pronounced in SLE patients (*r* = 0.3172; *p* < 0.0001, Figure 5) than in healthy controls (*r* = 0.2122; *p* = 0.0035, data not shown). Interestingly, most of the SLE patient sera with the highest titre of anti-HSA IgG also had the highest titre of anti-BSA IgG, which was not the case in the healthy control with the highest levels of anti-HSA IgG. Three SLE patients had both, high titres of anti-HSA IgG and high titres of anti-BSA IgG, and one patient (with the second highest antibody level) had only low anti-BSA IgG.

**Figure 6:**
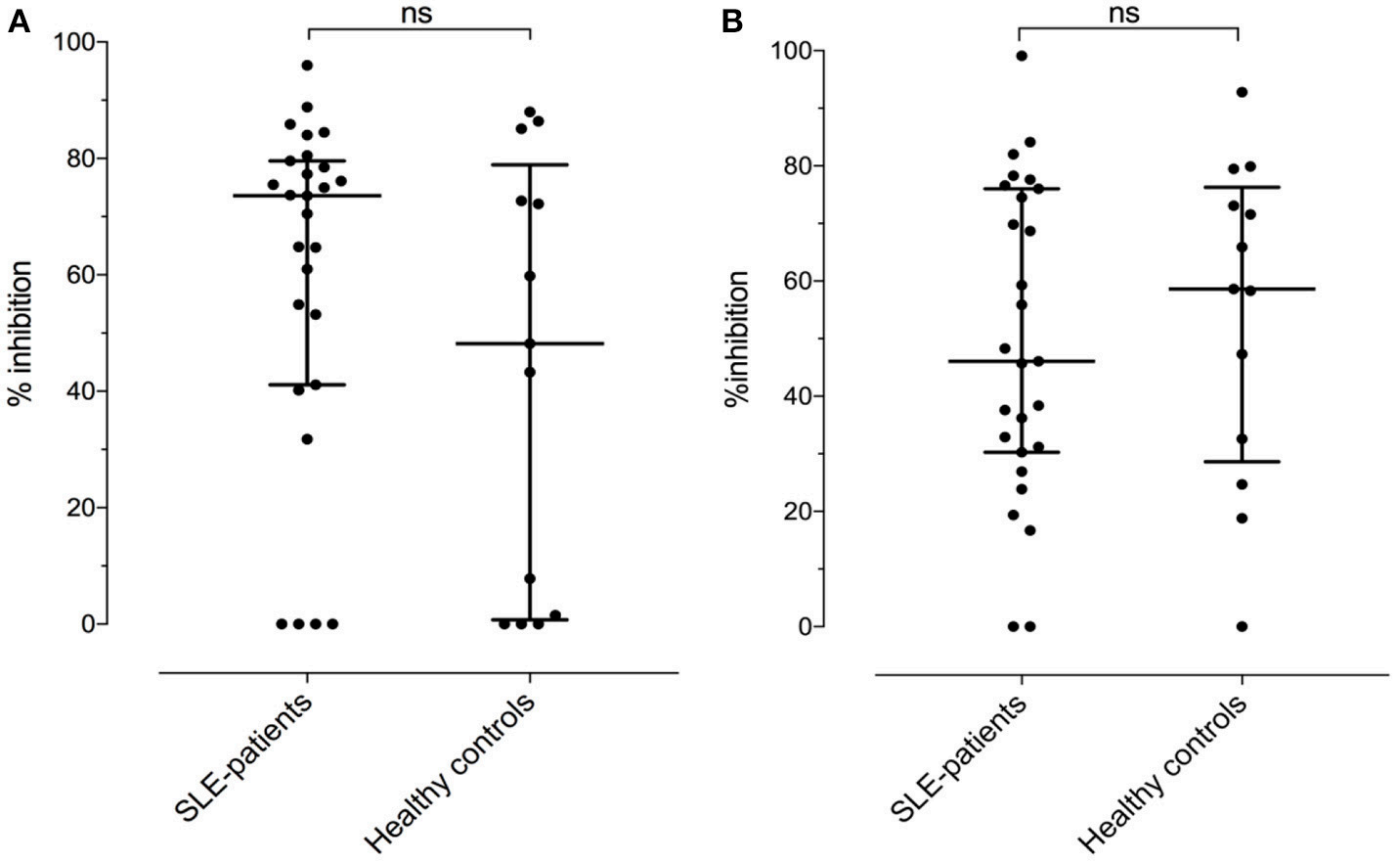
**(A)** Relative inhibition (percentage) of anti-HSA IgG by the presence of an excess in fluid phase BSA [median of SLE patients: 73.6 vs. healthy controls: 48.2, not significant (Mann–Whitney)]. (**B)** Relative inhibition (percentage) of anti-BSA IgG by the presence of an excess in fluid phase HSA [median of SLE patients: 46.1 vs. median of healthy controls: 58.6, not significant (Mann–Whitney)].

Assuming the occurrence of cross-reactive antibodies, we next tested whether anti-BSA IgG could be inhibited by the presence of fluid phase HSA, or anti-HSA IgG by BSA in 27 SLE patients and 13 healthy controls with double positivity. Overall, the median inhibition of anti-BSA IgG by HSA was 52% (range 0–99%). The median inhibition in SLE patients numerically was slightly less pronounced (46%) than in the healthy controls (59%) but this difference was not significant (*p* = 0.6482, Figure 6B). Similarly, the median inhibition of anti-HSA IgG by BSA was 68% (range 0–96%) with the median inhibition being more pronounced in SLE patients (74%) than in healthy controls (48%) (Figure 6A). Again, this difference was not significant (*p* = 0.2751). The relative inhibition of anti-BSA IgG by fluid phase HSA and of anti-HSA IgG by fluid phase BSA was dose dependent.

Taken together, these data suggest that anti-albumin antibodies are at least partially cross-reactive across species (human-bovine).

## Discussion

This is the first systematic analysis of the occurrence of IgG antibodies against HSA, the most abundant protein in plasma, and their association with clinical manifestations in patients with SLE. Anti-HSA IgG antibodies were found to occur as monomeric and as complexed antibodies. In spite of being rather rare, in patients with SLE these anti-HSA IgG correlated with overall disease activity, anti-dsDNA antibodies, anti-C1q antibodies and a history of arthritis as shown in a large and well-defined cohort of SLE patients. We also found that anti-HSA IgG correlate with levels of anti-BSA IgG, which was more pronounced in SLE patients than in controls. Among other reasons for this association, this observation could be explained by a possible cross-reactivity as we found a partial inhibition of anti-HSA IgG by the presence of BSA and vice versa.

Thus far, antibodies targeting HSA were scarcely described. Studies on anti-HSA antibodies were performed in small groups of patients, particularly in patients with liver diseases (liver cirrhosis, hepatitis B virus infection). In these patients, anti-HSA antibodies were found to primarily belong to the IgM class and to be complexed with HSA (21, 22). Another smaller study on patients suffering from familial dysautonomia, a congenital disorder of the autonomic nervous system, found an increased level of antibodies against BSA as well. Due to the extensive homology between BSA and HSA, the study also addressed the occurrence of anti-HSA that were found to belong to the IgM and IgG classes. In addition, a correlation between both, anti-BSA and anti-HSA, was found suggesting that anti-HSA could be induced by bovine milk antigens and recognized by antibodies binding to antigenic epitopes common in BSA and HSA (23).

Independently, glycosylated proteins have been described to play a role in the development of autoantibodies (24–26). In this context, Gregor et al. screened patients with insulin-dependent diabetes for the presence of antibodies against glycosylated albumin. While such a type of antibodies could not be detected, again IgM antibodies against unmodified HSA were identified (27).

In accordance with the results of previous studies, we also identified antibodies targeting BSA and investigated whether there was a relationship between anti-HSA IgG and anti-BSA IgG in SLE. Besides the correlation between anti-HSA and anti-BSA IgG, which was more pronounced in SLE patients, we could also demonstrate a partial inhibition of anti-HSA IgG by the presence of BSA and vice versa in individuals with double positivity for anti-HSA and anti-BSA IgG. The reasons for this partial cross-reactivity in our patients remain elusive and our data clearly do not provide a definite explanation, in particular because anti-BSA IgG were not increased in SLE patients. More complex experimental studies are required to establish or abandon a direct relationship between the two. One hypothesis could be that anti-HSA and anti-BSA IgG both are the result of epitope spreading, and that BSA can trigger or exacerbate the immune response against HSA. Such a hypothesis has already been formulated in previous studies. Another hypothesis is based on meat and diary products in general that have been described as a possible contributor to inflammatory responses, especially with regard to arthritis in patients with rheumatic diseases. Based on both, animal and human studies, it is assumed that proteins containing high levels of phenylalanine and tyrosine, such as found in beef and diary products, may modulate SLE symptoms, but data on the role of these proteins are controversial (28, 29). With regard to our study it is of interest to note that albumin also contains aromatic amino acids.

Using *in vivo* models and *in vitro* experiments, BSA has been shown to be involved in the development of arthritis due to an interaction with synoviocytes, in animals and in humans (30–34). Moreover, by means of a sequence alignment analysis, Pérez-Maceda et al. demonstrated that specific residues of BSA, which differed clearly from the corresponding fragment in HSA, were highly homologous with human collagen type I, C1q and vitamin D binding protein (16). Furthermore, representative sera of patients were found to have specific antibodies against BSA. Thus, it was assumed that the pathogenic mechanism of BSA in arthritis is explained by molecular mimicry.

Furthermore, in multiple sclerosis patients an association between anti-BSA antibodies and myelin basic protein has been reported by Winer et al. that is based on a structural homology between the two (17). The authors discussed an etiological role for BSA in the pathogenesis of multiple sclerosis comparable to studies with insulin dependent diabetic patients (IDDM) in which it was hypothesized that antibodies against food-derived BSA result in a cross-reactivity and consecutive destruction of beta cells (15, 35). Again, this view is controversial. More recent studies found no clear evidence for this hypothesis and suggest that the development of anti-BSA is part of a more general immune response against foreign proteins (36–38).

Last, as demonstrated *in vivo*, the intravenous injection of cationic BSA (c-BSA) can cause lesions of membranous nephropathy due to the deposition of immune complexes containing anti-BSA IgG and complement component C3 (39–41). Importantly, a more recent study by Debiec et al. could validate in four children with early-childhood membranous nephropathy a pathogenic role of c-BSA by the detection of high levels of anti-c-BSA IgG antibodies, suggesting that food-derived BSA may lead to glomerular lesions (18). In this context, it is noteworthy that in our study 11 of the 20 SLE patients with the highest anti-BSA IgG levels had a history of proliferative lupus nephritis [6 × class IV, 4 × class III, 1 × class II based on the International Society of Nephrology/Renal Pathology Society (ISN/RPS) classification of lupus nephritis (42)] vs. 45 of the remaining 160 patients (*p* = 0.0206, two tailed Fisher's exact test). If this observation can be confirmed it might be of relevance with regard to recommendations on diet in SLE patients, especially for patients with lupus nephritis.

Taken together, considering food-derived BSA as a causative factor for the association between anti-HSA and anti-BSA remains speculative and our observational data do not provide direct evidence for it. Other mechanisms might explain the association between anti-HSA and anti-BSA IgG as observed in our patients. For example, epitope spreading has been well-described to lead to autoantibody diversification in experimental models of SLE. Applying this concept to our observations, it is also conceivable that both types of antibodies (anti-HSA IgG and anti-BSA IgG) are the result of antibody spreading without any sequential relationship between the two (43–45).

In addition, our study has other limitations. Despite having analyzed a large cohort of patients, further studies are needed for the validation of our results, in particular with regard to the association between anti-HSA IgG and SLE disease activity. As we only analyzed SLE patients and matched healthy controls, we cannot estimate whether the occurrence of anti-HSA IgG is lupus-specific. For this, studies on patients with other rheumatic diseases (including rheumatoid arthritis and other connective tissue disorders) will be required, ideally using matched controls.

Second, although we used highly pure HSA as a reagent in our assays and in spite of attempts to address the topic by using different experimental techniques, we cannot fully exclude that impurity of the albumin preparations (HSA as well as BSA) might have partially influenced our signals. For example, pretreatment of HSA with increasing concentrations of DNAse led to a maximum drop of signal intensity of about 20% in one of the SLE sera.

Third, as anti-HSA IgG autoantibodies target a soluble antigen that is abundant in human plasma, an obvious question was whether our assay was able to detect all or at least a relevant and proportional part of the autoantibodies occurring *in vivo* (21). Therefore, our study included efforts to (i) analyze how much of the detected signal in our assay was the result of immune-complexes (vs. monomeric autoantibodies), and (ii) whether we missed a relevant part of autoantibodies that in fact were complexed and thus could not be detected in the ELISA. While the signal in our assay indeed largely consisted of complexed IgG, in SLE patients we could also detect anti-HSA IgG signals in the range of monomeric IgG suggesting that the signal consisted of either free autoantibodies and/or very small complexes. In addition, we analyzed the occurrence of albumin (HSA) -IgG complexes comparing SLE patients with healthy donors being matched for anti-HSA and anti-BSA IgG levels with a focus on individuals with low or even negative antibody levels. Interestingly, SLE patients tended to have more serum albumin (HSA)-IgG complexes than their matched controls, but we could not observe striking differences. This observation suggests that we did not miss large parts of the anti-HSA antibodies. It is important to note that albumin (HSA)-IgG complexes could also be detected in patients that were negative for anti-HSA and anti-BSA IgG respectively which might indicate, that the occurrence of these complexes is at least partially unspecific or due to low affinity interactions. However, we cannot exclude the possibility that in fact most anti-HSA autoantibodies are rapidly cleared by the reticuloendothelial system once being bound to their target. To overcome this problem, studies focusing on autoantibody-producing cells would be required. Last, our study was limited to the determination of IgG anti-HSA and did not include the analyses of other immunoglobulin classes. Here, beyond the determination of IgM as outlined above, IgA antibodies might be of particular interest. Increased levels of IgA as well as immune complexes containing IgA were described to play a pathogenic role based on the observation of increased cartilage erosion and the occurrence of erosive arthritis in patients with high levels of IgA, especially in patients with rheumatoid arthritis (46, 47).

In conclusion, anti-HSA IgG were found to have an increased prevalence in SLE patients and were associated with SLE disease activity. Furthermore, anti-HSA IgG were found to correlate with levels of IgG antibodies against bovine serum albumin. Further studies are required to determine the role of anti-HSA and anti-BSA IgG in SLE, in particular with regard to their association with lupus nephritis.

## Author contributions

MT directed the project, conceived of the presented idea and planned the experiments. JN aided to carried out the experiments and measurements with DD, processed the experimental data, performed the analysis, drafted and wrote the manuscript and designed the figures. CC (Geneva) and CR (Lausanne) provided data of the SLE patients who were included in SSCS. UH-D provided data of the SLE patients who were included in Bern. DD performed the experiments and measurements. LS assisted with measurements of titres of anti-DNA-antibodies by ELISA. JF contributed with knowledge regarding calculations with Scatchard Plot Analysis and provided data of the study cohort regarding lupus nephritis. All authors commented on the manuscript.

### Conflict of interest statement

The authors declare that the research was conducted in the absence of any commercial or financial relationships that could be construed as a potential conflict of interest.
