# Supplementary material for: Autoantibodies Against Albumin in Patients With Systemic Lupus Erythematosus
Source: Front Immunol. 2018 Oct 2;9:2090. doi: 10.3389/fimmu.2018.02090 (PMC6176020; doi:10.3389/fimmu.2018.02090)
Supplement: Supplementary file 1 [file Data_Sheet_1.docx]

## Supplement Figure 1


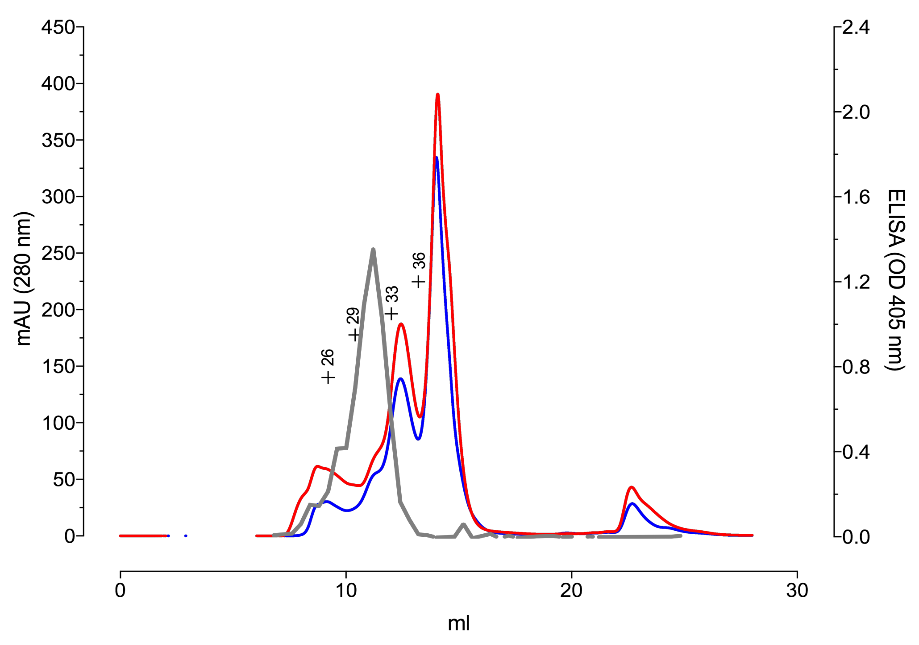
A

B

HSA 26 29 33 36 L 26 29 33 36


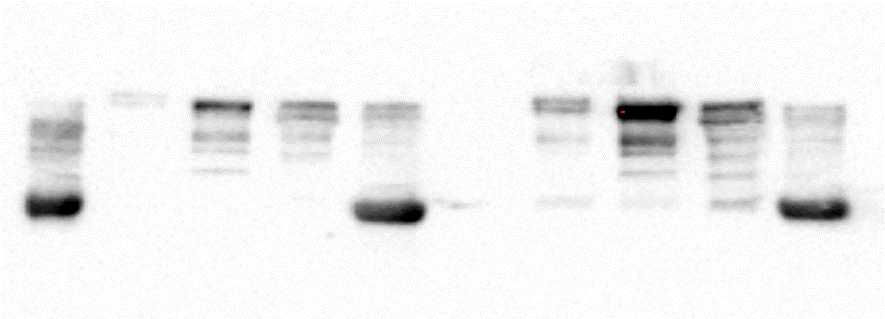


---------------SLE 4--------------- ------Healthy control 1------

C

HSA 26 29 33 36 L 26 29 33 36


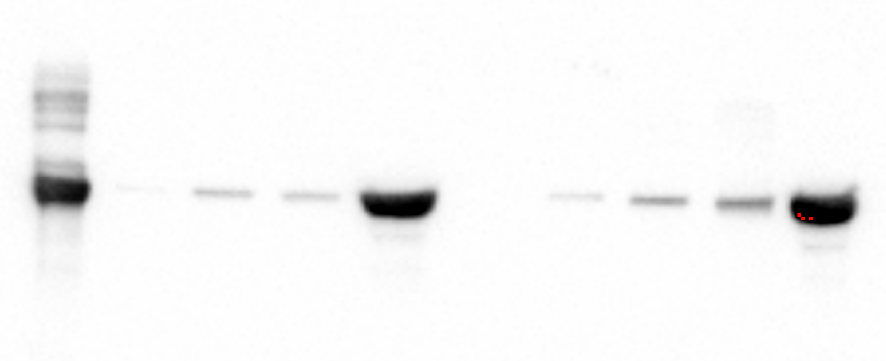


-------------SLE 4------------------ -----Healthy control 1----

**Suppl.Fig.1A-C.**

**Suppl.Fig.A**

Serum profiles of SLE patient 4 (red line) and healthy control 1 (blue line) separated by FPLC. The grey line represents the signal for the detection of albumin (HSA)-IgG complexes of the healthy control 1 as quantified by ELISA. Numbers (+26,+29,+33,+36) indicate the localisation of serum fractions for the determination of the presence of albumin by Western Blot as used in part B of the figure.

**Suppl.Fig.1B**

Detection of albumin by Western Blot in non-reduced FPLC fractions of SLE patient 4 and healthy control 1 as indicated in Suppl.Fig. 1A.

**Suppl.Fig.1C**

Detection of albumin by Western Blot in reduced FPLC fractions of SLE patient 4 and healthy control 1.

**Immunoblot** **SDS-Page** **Immunoblot**

IgG on reduced HSA IgG on unreduced HSA

SLE SLE NHS No red. marker unred. SLE SLE NHS

### #4 #2 #1 serum HSA HSA #4 #2 #1


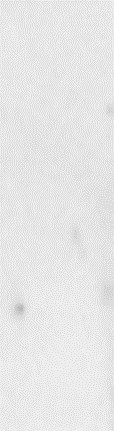

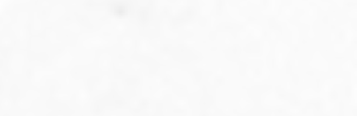

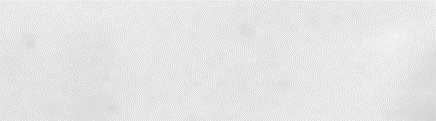

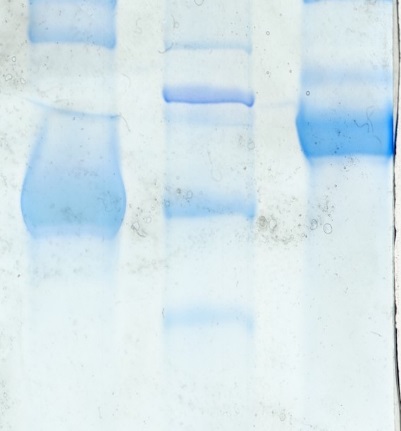

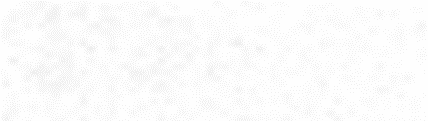

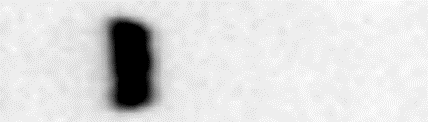

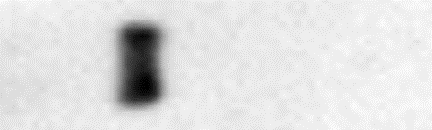

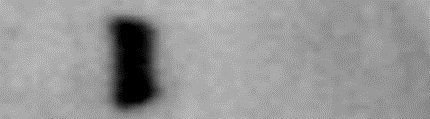


100 kDa –

75 kDa –

50 kDa –

37 kDa –

**Suppl.Fig.2**

The Figure shows serum IgG binding to purified reduced and unreduced HSA respectively as determined by Western Blot. Lanes 1-3 show a positive binding of serum IgG to reduced HSA in anti-HSA IgG positive sera (SLE 4, SLE 2 and healthy control 1); lane 4 demonstrates a lack of signal in the absence of serum (negative control). No serum IgG binding of the same sera could be detected when using unreduced HSA (lanes 8-10). Lanes 5-7 show the corresponding protein bands of reduced HSA, the marker molecules and unreduced HSA, respectively.
